# IL‐23 plays a significant role in the augmentation of particulate matter‐mediated allergic airway inflammation

**DOI:** 10.1111/jcmm.17475

**Published:** 2022-07-08

**Authors:** Hyun Seung Lee, Heung‐Woo Park

**Affiliations:** ^1^ Biomedical Research Institute Seoul National University Hospital Seoul Korea; ^2^ Department of Internal Medicine Seoul National University Hospital Seoul Korea; ^3^ Department of Internal Medicine Seoul National University College of Medicine Seoul Korea

**Keywords:** asthma, house dust mite, immune response, interleukin 23, particulate matter

## Abstract

It has been recently that particulate matter (PM) exposure increases the risk and exacerbation of allergic asthma. However, the underlying mechanisms and factors associated with increased allergic responses remain elusive. We evaluated IL‐23 and IL‐23R (receptor) expression, as well as changes in the asthmatic phenotype in mice administered PM and a low dose of house dust mite (HDM). Next, changes in the phenotype and immune responses were evaluated after intranasal administration of anti‐IL‐23 antibody during co‐exposure to PM and low‐dose HDM. We also performed in vitro experiments to investigate the effect of IL‐23. IL‐23 expression was significantly increased in Epcam+CD45− and CD11c+ cells, while that of IL‐23R was increased in Epcam+CD45− cells only in mice administered PM and low‐dose HDM. Administration of anti‐IL‐23 antibody led to decreased airway hyperresponsiveness, eosinophils, and activation of dendritic cells, reduced populations of Th2 Th17, ILC2, the level of IL‐33 and granulocyte‐macrophage colony‐stimulating factor (GM‐CSF). Inhibition of IL‐23 in PM and low‐dose HDM stimulated airway epithelial cell line resulted in decreased IL‐33, GM‐CSF and affected ILC2 and the activation of BMDCs. PM augmented the phenotypes and immunologic responses of asthma even at low doses of HDM. Interestingly, IL‐23 affected immunological changes in airway epithelial cells.

## 1 INTRODUCTION

Bronchial asthma is a chronic respiratory allergic disease with high epidemiological importance. It is understood that a combination of genetic and environmental factors might be involved in its pathophysiology.^1^ Particulate matter (PM) has recently emerged as an important causative agent of bronchial asthma.^2^ In particular, it has been shown that when a patient with asthma is exposed to PM, lung function decreases, symptoms are worsened, and bronchial control is more difficult.^3^ According to previously reported epidemiological studies, such PM is known to increase the morbidity of allergic diseases by causing barrier dysfunction, oxidative stress and immune imbalance of airway epithelial cells following exposure to various allergens.^4^, ^5^

In ovalbumin (OVA) asthma mice, PM was demonstrated to enhance the allergic inflammatory response.^6^ It has also been reported that the number of Th2 and Th17 cells was increased by diesel exhaust particle (DEP) in a mouse model exposed to house dust mite (HDM) antigen, with interleukin 33 (IL‐33) playing an important role in this process.^7^, ^8^ However, studies on animal models and the cellular mechanisms underlying the increased susceptibility to allergens and on factors that could potentially inhibit them are still insufficient.

Airway epithelial cells are innate cells well‐known to come first in contact with several environmental factors.^9^ In addition, it has also known that the crucial site of accumulation of inhaled PM is the airway epithelium, which provides a chemical, immunological and mechanical protective barrier against environmental irritants.^10^ In addition, innate cytokines such as thymic stromal lymphopoietin (TSLP), IL‐25 and IL‐33 secreted from airway epithelial cells promote and amplify Th2 responses through innate cells (ILC2, basophils, mast cells and eosinophils) and induce dendritic cells to lean towards pro‐Th2 phenotype.^11^, ^12^

Interleukin 23 (IL‐23) is known to be mainly secreted by antigen‐presenting cells and has been shown to be involved in adaptive immune responses such as Th17 activation.^13^ However, it was recently suggested that it is also expressed in airway epithelial cells, playing an important role in the occurrence of asthma by acting on the innate and adaptive immune responses.^12^ Moreover, it is known that the secretion of IL‐23 from airway epithelial cells can regulate the expression of innate cytokines such as IL‐33 and TSLP.^14^, ^15^

In this study, we intend to investigate whether PM exposure and low concentrations of allergens can enhance asthma and to identify the role of IL‐23 in adaptive and innate immune‐related mechanisms in this process.

## 2 MATERIALS AND METHODS

### 2.1 Animal

Six‐week‐old female BALB/C mice (18–20 g) were purchased from Orient Bio. All experiments were performed with the approval of the Institutional Animal Care and Use Committee of the Institute of Laboratory Animal Resources at Seoul National University (SNU‐190922‐2) and Seoul National University Hospital (SNUH‐IACUC 20‐0044‐S1A0).

### 2.2 Preparation of particulate matter (PM_10_ )

We collected PM samples in the Seoul, Korea. This PM sample was representative of urban traffic pollution. Particulates were released from filters (<10 μm); the samples were extracted and sonicated in phosphate‐buffered saline (PBS). This PM10 has been contained with a variety of metals, notably including Si (41%), Fe (16.9%), Al (14.9%), Ca (10.1%), K (7.63%), Mg (2.27%) and S (1.28%). In addition, the concentration of water‐soluble ions is SO_4_^2−^ (2000 mg/kg) and NO_3_^−^ (400 mg/ml). These analyses were conducted by Korea Institute of Science and Technology and Korea Research Institute of Chemical Technology.

### 2.3 Mouse experiments

Particulate matter (10 mg) was dissolved in 10 ml PBS containing 0.1% Dimethyl sulfoxide (DMSO). This stock of PM (1 mg/ml) was suspended in PBS for administration of mice (20–100 μg/mouse). Mice were instilled PM or low dose of Dp (10 μg/mouse, Dermatophagoides pteronyssinus; Dp 1 1.88 μg/mg protein, PROLAGEN) with/without anti‐IL‐23p19 antibody (0.5 μg/mouse, R&D systems) by intranasal injection. Goat IgG Ab was administrated in without anti‐IL‐23p19 antibody‐treated groups as control.

### 2.4 Airway hyperreactivity

Measurement of dynamic resistance was performed using a Flexivent system (Scireq). Mice were anaesthesia with ketamine (90 mg/kg body weight) and xylazine (10 mg/kg body weight), tracheostomized and connected to the flexivent ventilator via a 19‐gauge cannula. Positive end‐expiratory pressure is 3 cm H_2_O. Measurement of airway resistance (cm H_2_O/ml/s) was determined using snapshot‐150 perturbation. MCh (acetyl‐b‐methylcholine chloride; Sigma‐Aldrich) provocation testing started with PBS, followed by MCh aerosols with increasing concentrations (0, 12.5, 25, 50 and 100 mg/ml). The graphs show values of 50 and 100 mg/ml.

### 2.5 Evaluation of DC activation in lung draining lymph node (LDLN)

Mice were sacrificed 24 h after last instillation. The LDLNs were removed and processed. Single cells stained with APC/cy7‐conjugated anti‐CD11c antibody (BD Biosciences), APC‐conjugated anti‐MHCII antibody (Thermo Fisher Scientific) and PE‐conjugated anti‐CD86 antibody (Thermo Fisher Scientific). The percentage of activated DCs was determined by flow cytometry as the fraction of MHCII+ or CD86+ in CD11c+ cells. Each sample was read on LSRII (BD Biosciences).

### 2.6 Histopathology

To evaluate and compare the severity and character of pathological changes in lung parenchyma, left lungs of mice were fixed in 10% neutral buffered formalin and embedded in paraffin, and 3‐mm sections were stained with haematoxylin and eosin, and for immunohistochemistry stain, the lung sections were incubated with as each primary antibody. Alpha‐smooth muscle actin (1:200, Abcam) was used as primary antibodies. As isotype control, anti‐rabbit IgG Ab was used. IHC stain pictures were taken in a digital image program (iSolution Lite) with Nikon light microscope. Slides were examined at 200× magnification.

### 2.7 ELISA assay, analysis of IL‐23, IL‐23R (Receptor), Th cells and innate lymphoid cells (ILCs) in lung tissues

For detection of proteins level in isolated one lobe of lung tissue, lungs (40 mg/each) were homogenized with PBS (0.5 ml) and then centrifuged at 12,000 *g* for 10 min, and supernatants were saved at −80°C. In the supernatants of crushed lungs, IL‐23, IL‐33, TSLP and GM‐CSF were measured using ELISA (R&D Systems) following the manufacturer's instructions. Der p specific IgE and IgG1 level in serum was also measured using enzyme‐linked immunosorbent assay, in accordance with the manufacturer's instructions. For detection of IL‐23 and IL‐23R expression in lung tissue, after the preparation of single cell, the cells were treated golgistop and were stained with Percp‐cy5.5‐conjugated anti‐CD45 (Thermo Fisher Scientific), APC/cy7‐conjugated anti‐CD11c (Thermo Fisher Scientific), BV421‐conjugated anti‐Epcam (Thermo Fisher Scientific) and PE‐conjugated anti‐IL‐23R (Thermo Fisher Scientific). For intracellular staining, cells were permeabilized (Cytofix/Cytoperm kit; BD Biosciences) and incubated with APC‐conjugated anti‐IL‐23p19 (Thermo Fisher Scientific). Each sample was read on LSRII (BD Biosciences). Type 2 innate lymphoid cells (ILC2) in lung were determined by flow cytometry. After the preparation of single cell, the cells were stimulated by PMA (100 ng/ml), ionomycin (1 μg/ml) and golgistop. Cells were stained with Percp‐cy5.5‐conjugated anti‐CD45 (Thermo Fisher Scientific). For detection of ILC2s and ILC3s, Lineage‐negative cells were gated as cells not expressing CD3, CD4, CD8, CD11b, CD11c, CD19, F4/80, FcεRI and CD49b, and PerCP‐conjugated ST2, APC‐conjugated ICOS also stained. For intracellular staining, cells were permeabilized (Cytofix/Cytoperm kit) and incubated with PE‐cy7‐conjugated anti‐IL‐13 (Thermo Fisher Scientific) or APC‐conjugated anti‐IL‐5 (BD Bioscience) or BV421‐conjugated anti‐IL‐17 (Thermo Fisher Scientific). For analysis of Th2,Th17 cells, BV711‐conjugated anti‐CD4 antibody was also used. For the analysis of data using flow cytometry, each sample was read at least 200,000 cells on LSRII and FACSymphony (BD Biosciences).

### 2.8 Evaluation of IL‐23 and IL‐23R in PM‐ or Dp‐stimulated MLE12 cells and the detection of IL‐23, IL‐33 and GM‐CSF in IL‐23 siRNA treated MLE12 cells

For detection of IL‐23 and IL‐23R expression, MLE‐12 cells (SV40‐transformed mouse‐derived alveolar epithelial cell line; American Type Culture Collection) were grown in Dulbecco's modified Eagle medium:Ham's F‐12 with 2% fetalbovine serum in a humidified atmosphere at 37°C with 5% CO2. The cells were then stimulated with low‐dose Dp (10 μg/ml) or PM (0.1 μg/ml) for overnight. The expression of IL‐23 was detected in cytosol using NE‐PER Extraction Reagent (Thermo Fisher Scientific) and IL‐23 ELISA kit (Biolegend). For expression of IL‐23R, cells were stained with PE‐conjugated anti‐IL‐23R (Thermo Fisher Scientific). For IL‐23 siRNA experiment, MLE12 cells were treated with IL‐23p19 siRNA (Santa Cruz, 30 nM/well) or normal control (NC, Santa Cruz) siRNA using transfectamine (Invitrogen) and after 5 hours, the cells were washed and stimulated low‐dose Dp (10 μg/ml) or PM (0.1 μg/ml) for overnight. The protein level of IL‐23, GMCSF in cytosol and IL‐33 in the nucleus was measured using NE‐PER Extraction Reagent (Thermo) and ELISA kit (R&D). For detection of intracellular IL‐33 expression from MLE‐12 cells, the cells were permeabilized and incubated with anti‐IL‐33 (1:100, R&D), 647‐conjugated anti‐goat IgG (1:500, Invitrogen). Each sample was read on LSRII (BD Biosciences).

### 2.9 Sorting of type 2 innate lymphoid cell (ILC2)

Mice were treated with rmIL‐33 (0.5 μg/mouse) for 1 week (Figure 6A). Lungs were harvested, and for sorting of ILC2s, lineage‐negative cells were gated as cells not expressing FITC‐conjugated CD3, CD4, CD8, CD11b, CD11c, CD19, F4/80, FcεRI and CD49b, and PE‐conjugated ST2, PE/cy7‐conjugated IL‐7R also stained. Several mice (8 mice) were pooled for sorting of ILC2s. FACSAriaII was used (BD Bioscience) for sorting of ILC2. Sorted lung ILC2 (5 × 10^4^/well) was expanded in vitro in 10% FBS RPMI complete medium containing IL‐2 (10 ng/ml, R&D Systems), IL‐7 (10 ng/ml, R&D Systems) and IL‐33 (10 ng/ml, R&D Systems) for 7 days.

### 2.10 Coculture of ILC2s or BMDCs with IL‐23 siRNA treated MLE12 cells

For indirect co‐cultivation of epithelial cell and ILC2 or BMDCs, to find out the effect of the secreted molecules from epithelial cell, 12‐well plate with transwell inserts (pore size: 0.4 mM) (Costar) was used to separate these two cells into two compartments. MLE12 cells also treat IL‐23p19 siRNA or NC siRNA and after 5 hours, the cells were washed and stimulated low‐dose Dp (10 μg/ml) or PM (0.1 μg/ml) for overnight. After washing, ILC2 was cultured together in the presence of transwell inserts in which MLE12 cells and ILC2 were placed in the upper and lower compartment. They were incubated for 24 h. The cells were treated golgistop 4 h before harvest; the cells were permeabilized (Cytofix/Cytoperm kit) and incubated with PE‐cy7‐conjugated anti‐IL‐13 (Thermo Fisher Scientific) or APC‐conjugated anti‐IL‐5 (BD Bioscience). For coculture with BMDCs, BMDCs were prepared following the method described previously.^16^ At Day 7, BMDCs were seeded in the lower well of Transwell inserts. MLE12 cells also treat IL‐23p19 siRNA or NC siRNA for 5 h and the cell was washed, and low‐dose Dp (10 μg/ml) or PM (0.1 μg/ml) for overnight. After washing, these upper wells placed in the lower wells with BMDCs. After 24 h of coculture, cells stained with APC/cy7‐conjugated anti‐CD11c antibody and APC‐conjugated anti‐MHCII antibody. Each sample was read on LSRII (BD Biosciences).

### 2.11 Analysis of data using flow cytometry

All data were analysed using FlowJo software (BD Biosciences).

### 2.12 Statistics

Results are expressed as means ± standard deviation, and statistically significant differences (*P* < 0.05) were determined using one‐way anova with Bonferroni post‐test for multiple comparisons. Statistical analyses were performed using GraphPad Prism (GraphPad Software 9.0.1).

## 3 RESULTS

### 3.1 Particulate matter induced allergic responses and expression of IL‐23 in low‐dose Dp‐instillated mice

**FIGURE 1:**
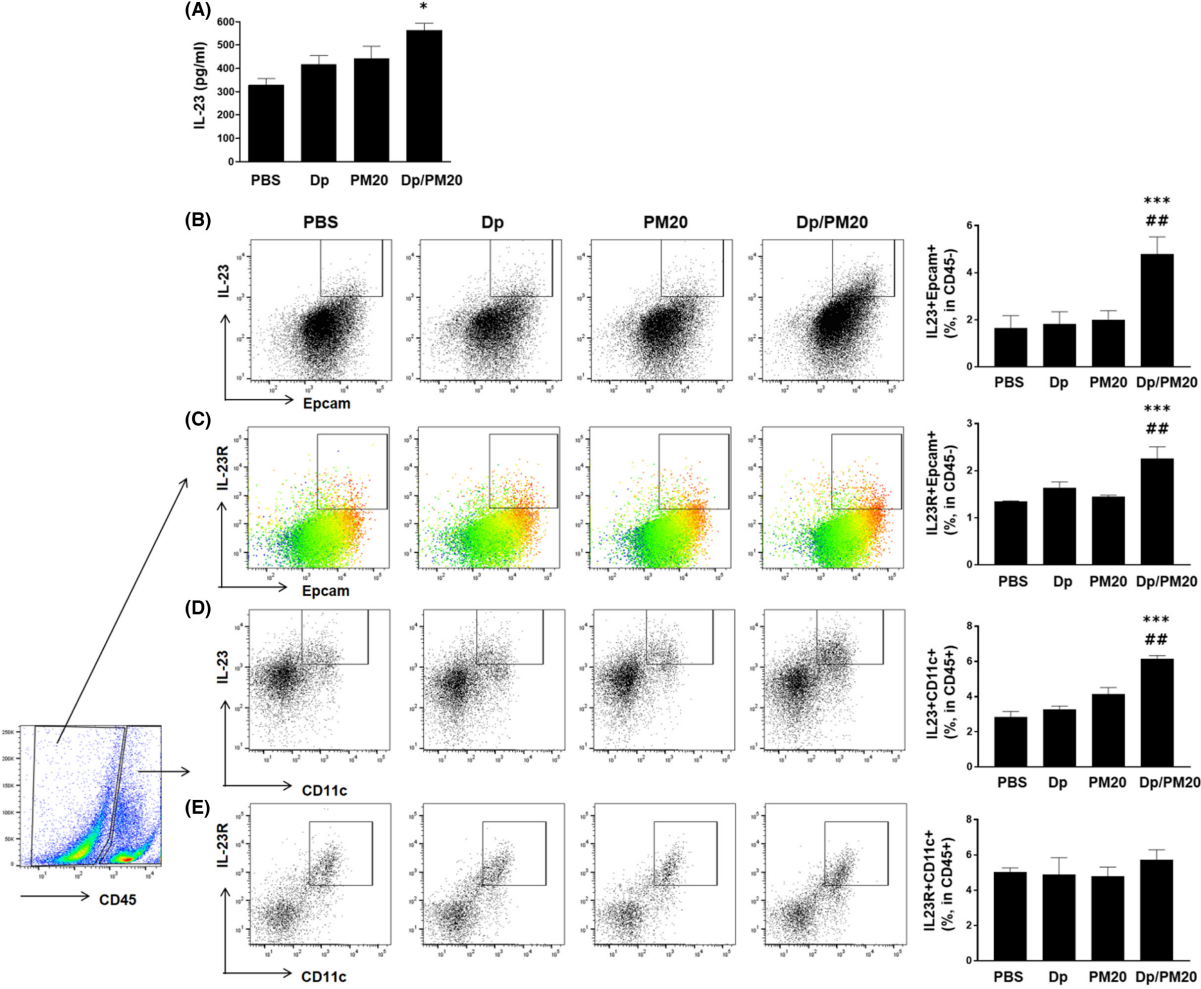
Expression of IL‐23 and IL‐23R in PM‐ and low‐dose Dp‐exposed mice. PM (20 μg) with low dose of Dp (10 μg) were instillated in mice on Days 1, 3, 5, 8, 10, 12 and 15 (4–5 mice in each group). 24 h after the last instillation, the level of IL‐23 was evaluated. The protein level of IL‐23 in crushed lung was detected using ELISA (A). The gating plots and frequencies of IL‐23 + Epcam+ (B) or IL‐23R + Epcam+ (C) in CD45− cells and IL‐23 + CD11c+ (D) or IL‐23R + CD11c + (I) in CD45+ cells (E) from lung tissue were detected using flow cytometry. PM: Particulate matter. IL‐23R: IL‐23 Receptor. Statistically significant (*P* < 0.05, *P* < 0.01, *P* < 0.001) differences from Dp group are represented by *, and differences from each dose of PM group are represented by #. Statistical analysis followed by one‐way anova with Bonferroni's multiple comparisons test

First, in order to investigate the effect of PM on the low dose of Dp exposure process, the results of administration by dose of PM, we found that in mice administered a combination of low dose of Dp and PM 20, bronchoalveolar lavage fluid (BALF) eosinophils, Dp‐specific IgG1 and the level of IL‐5 and IL‐13 from BALF was increased compared with that in mice administered Dp or PM alone (Figure S1A–D). Hence, we determined the concentration of PM that could amplify this response even at a low dose of exposure to Dp. We next observed that the secretion of IL‐23 was increased in BALF of mice administered both Dp (10 μg) and PM (20 μg) compared with that in the low‐dose Dp group (Figure 1A). Using flow cytometry, we found that the expression of intracellular IL‐23 and surface IL‐23R in epcam+cd45− (epithelial cell marker) cells increased significantly in the combined‐treated group compared with that in the PM or low‐dose Dp group (Figure 1B,C). Compared with that in the low‐dose Dp group, the intracellular level of IL‐23 was found to be increased in cd11c+ cells (Figure 1D), whereas the expression of IL‐23R did not show any difference among the groups (Figure 1E).

### 3.2 Inhibition of IL‐23 reduced the increase in allergic responses, AHR and the expression of α‐SMA in PM‐exposed and low‐dose Dp‐instillated mice

**FIGURE 2:**
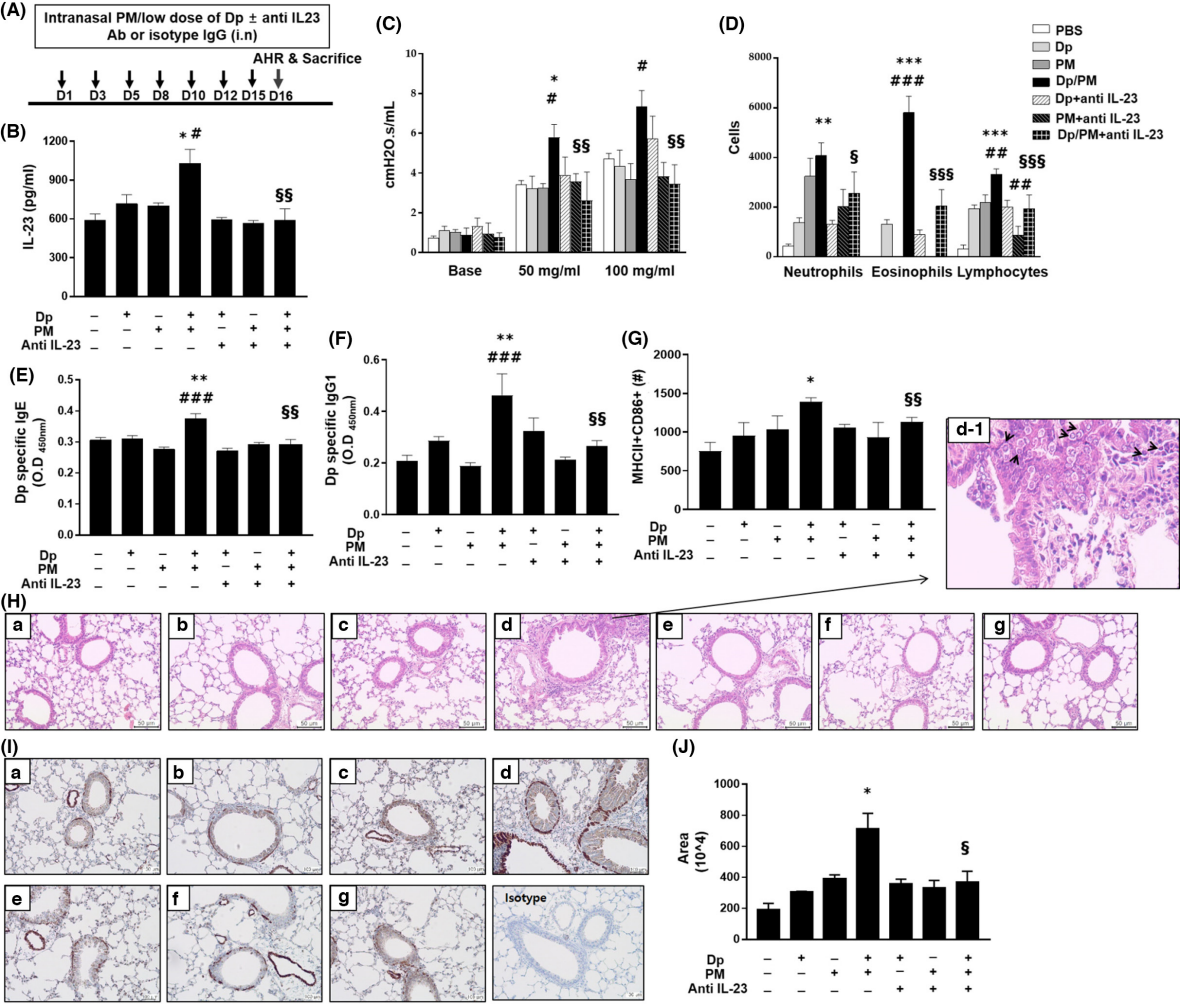
Evaluation of asthmatic phenotypes. Experimental protocol (5–6 mice in each group) (A). The protein level of IL‐23 in crushed lung was detected using ELISA (B). Airway hyperresponsiveness. (Y‐axis of graph represents cmH2O.s/ml) (C). The numbers of inflammatory cells including neutrophils, eosinophils and lymphocytes in BALF (D). Serum Dp‐specific IgE and IgG1 were evaluated after the last challenge (*Y*‐axis of graph represents O.D: optical density) (E,F). The numbers of MHCII+ CD86+ cells in CD11c+ DCs from lung draining lymph node were determined using flow cytometry (G). Haematoxylin and eosin stain in lung tissue after the last instillation (×200) (H). All scale bars represent 50 μm. (a: PBS, b: Dp, c: PM, d: Dp/PM, e: Dp + anti‐IL‐23 Ab, f: PM + anti‐IL‐23 Ab, g: Dp/PM + anti‐IL‐23 Ab). Black arrows indicate eosinophils (d‐1). Immunohistochemisty stain of alpha‐smooth muscle actin (α‐SMA) in lung tissue after the last instillation (I, ×200). All scale bars represent 100 μm. Comparison of area of α‐SMA (J). Area% was quantified using ImageJ software (4–5 airways in 3–4 mice per each group). PM: Particulate matter. Statistically significant (*P* < 0.05, *P* < 0.01, *P* < 0.001) differences from Dp group are represented by *, differences from PM group are represented by #, and differences from Dp/PM group are represented by §. Statistical analysis followed by one‐way anova with Bonferroni's multiple comparisons test

Similar to the previous experiment, PM, low‐dose Dp and anti‐IL‐23 antibody were administered in the mouse model to determine their effect on the allergic response (Figure 2A). First, we observed the changes in the secretion of IL‐23 following administration of PM, low‐dose Dp and anti‐IL‐23 antibody (Figure 2B). We found that in mice administered with both PM and low‐dose Dp, AHR, the increased numbers of eosinophils, neutrophils and lymphocytes in BALF were reduced following the administration of an anti‐IL‐23 antibody compared with that in the PM and low‐dose Dp group (Figure 2C,D). In addition, the increase in serum Dp‐specific IgE and IgG1 levels and activation of DCs from LDLN in PM‐ and low‐dose Dp‐treated mice was also reduced following the administration of an anti‐IL‐23 antibody (Figure 2E–G). Haematoxylin and eosin staining results revealed an increased number of infiltrating inflammatory cells including eosinophils around the airway in PM‐ and low‐dose Dp‐treated mice compared with that in the low‐dose Dp group. We further noted that when an anti‐IL‐23 antibody was administered, these inflammatory cells were decreased (Figure 2H).

As one of the mechanisms for the increase in airway hyperresponsiveness, we tried to confirm the change in the expression of α‐SMA.^17^ To evaluate this, we performed IHC staining in lung tissues and found that the expression of α‐SMA around the airways was increased when PM and low‐dose Dp were administered, whereas it was decreased when anti‐IL‐23 antibody was administered (Figure 2I,J).

### 3.3 Inhibition of IL‐23 reduced the increase in Th2, Th17 and ILC2 cells in PM‐exposed and low‐dose Dp‐instillated mice

**FIGURE 3:**
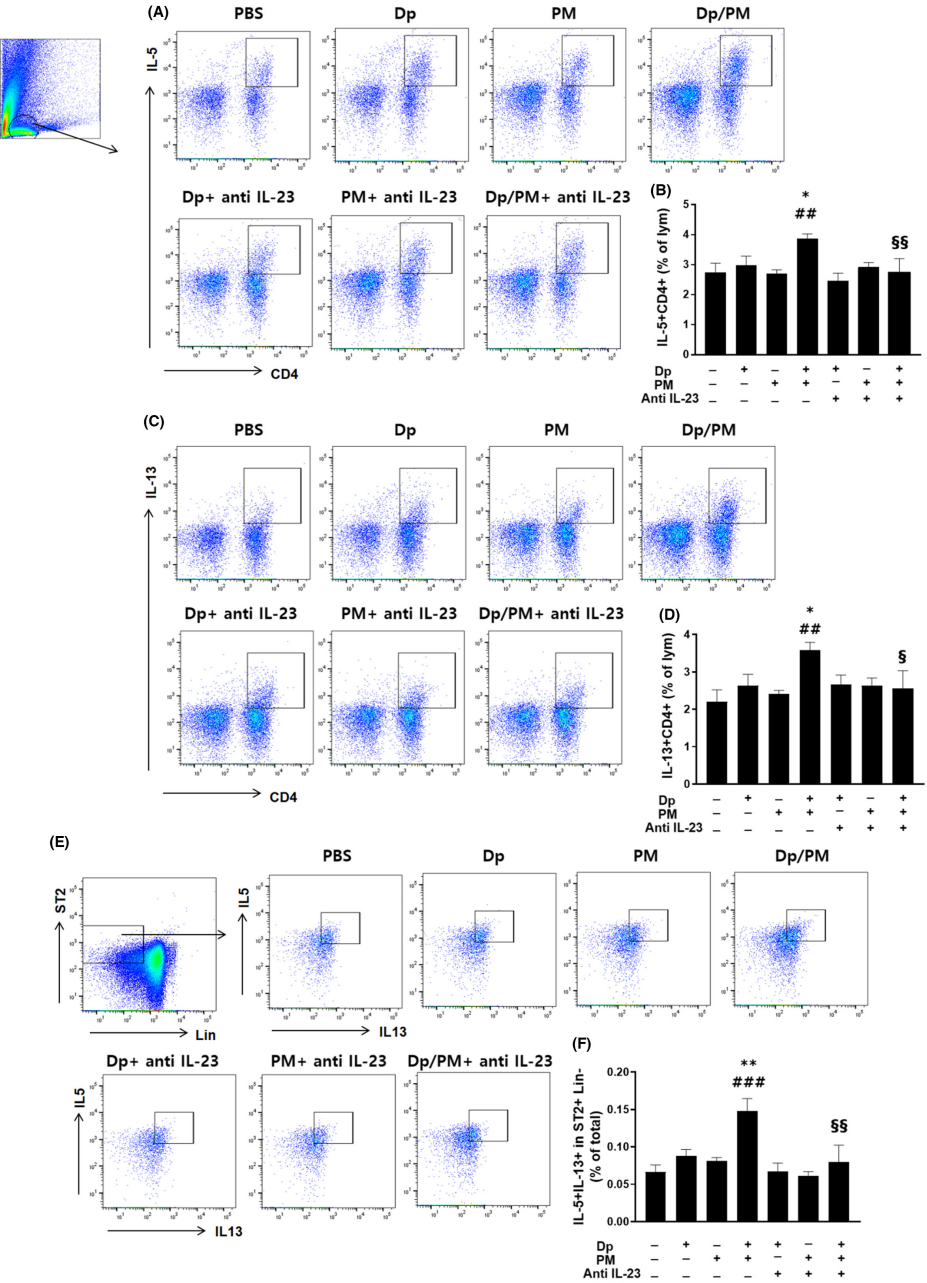
Population of Th2 cells and ILC2. The gating plot and the frequency of IL‐5+ in CD4+ T cells from lymphocytes in lung cells (A, B). The gating plot and the frequency of IL‐13+ in CD4+ T cells from lymphocytes in lung cells (C, D). The gating plot and the frequency of IL‐13 + IL‐5+ type 2 innate lymphoid cells (CD45+ Lineage‐ ST2+) in lung cells (E, F). PM: Particulate matter. Statistically significant (*P* < 0.05, *P* < 0.01, *P* < 0.001) differences from Dp group are represented by *, differences from PM group are represented by #, differences from Dp/PM group are represented by §. Statistical analysis followed by one‐way anova with Bonferroni's multiple comparisons test

**FIGURE 4:**
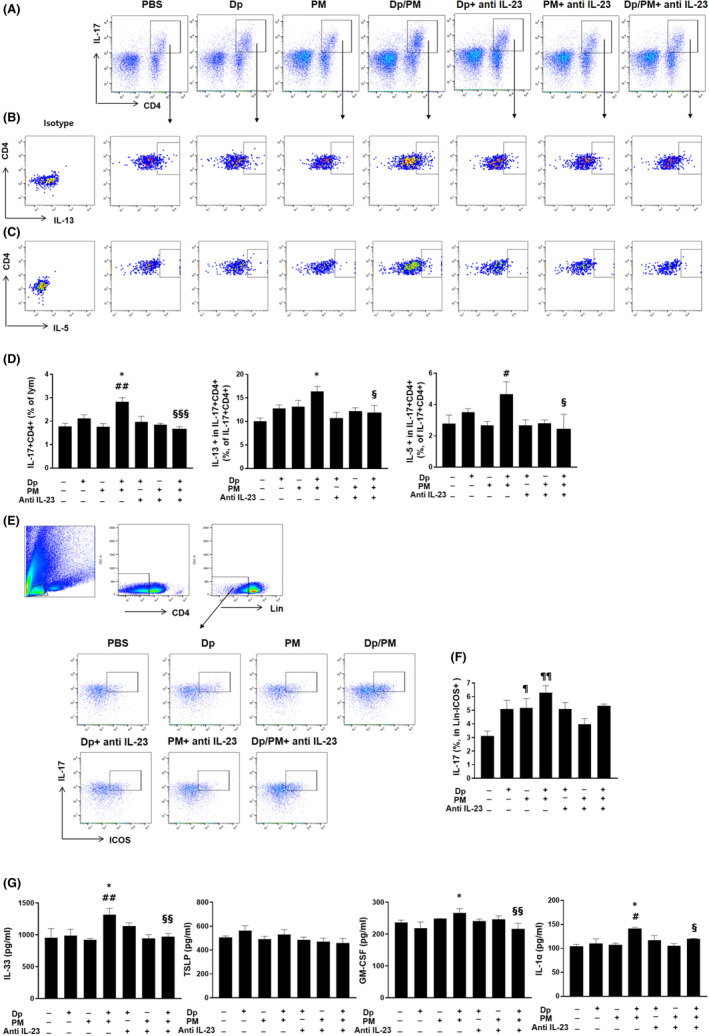
Population of Th17 cells, ILC3, the level of innate cytokines. The gating plot of IL‐17 + CD4+ T cells from lymphocytes and IL‐13 or IL‐5‐producing from IL‐17 + CD4+ cells in lung cells (A–C). The frequency of IL‐17 + CD4+ T cells from lymphocytes and IL‐13 or IL‐5‐producing from IL‐17 + CD4+ cells in lung cells (D). The gating plot and frequency of IL‐17A‐producing type 3 innate lymphoid cells (CD45+ Lineage‐ ICOS+) in lung cells (E, F). IL‐33, TSLP, GM‐CSF and IL‐1α levels in crushed lung (G). PM: Particulate matter. Statistically significant (*P* < 0.05, *P* < 0.01, *P* < 0.001) differences from Dp group are represented by *, differences from PM group are represented by #, differences from Dp/PM group are represented by §, and differences from PBS group are represented by ¶. Statistical analysis followed by one‐way anova with Bonferroni's multiple comparisons test

We also evaluated the changes in the numbers of Th cells, Th2 cells (IL‐5‐ or IL‐13‐producing CD4+ lymphocytes), Th17 cells (IL‐17‐producing CD4+ lymphocytes), and IL‐5+ or IL‐13+ type 2 innate lymphoid cells (ILC2), which are in ST2 + Lin‐.^18^ We found that their numbers increased following the administration of PM and low‐dose Dp compared with low‐dose Dp only, whereas they significantly decreased when anti‐IL‐23 antibody was administered (Figures 3A–F and 4A,D). Dual‐positive Th2/Th17 cells have recently been suggested to play an important role in asthma.^19^ We further observed that the levels of IL‐13 or IL‐5 secreted from IL‐17+ CD4+ cells were also increased in PM‐ and low‐dose Dp‐treated mice compared with that in the low‐dose Dp or PM group, whereas they significantly decreased when anti‐IL‐23 antibody was administered (Figure 4B–D). In the case of ILC3 (IL‐17 in ICOS;Inducible T‐Cell Costimulator + Lin‐),^20^ this increase was observed in the PM alone group when compared with the PBS group; combined administration of PM and low‐dose Dp did not lead to a significant increase compared with that in the PM or the low‐dose Dp alone groups (Figure 4E,F). To explain the mechanisms underlying the reduction in ILC2 levels and activation of DCs from LDLN through the inhibition of IL‐23, we measured the changes in IL‐33 levels, TSLP, granulocyte‐macrophage colony‐stimulating factor (GM‐CSF) and IL‐1α. We found that the protein levels of IL‐33, GM‐CSF and IL‐1α in crushed lung supernatants were significantly increased upon exposure to PM and low‐dose Dp, whereas they were significantly reduced in anti‐IL‐23 Ab‐treated mice. However, the protein levels of TSLP in the crushed lung supernatants did not change (Figure 4G).

### 3.4 Expression of IL‐23, IL‐23R, GM‐CSF and IL‐33 was increased in PM‐ and low‐dose Dp‐stimulated MLE‐12 cells

**FIGURE 5:**
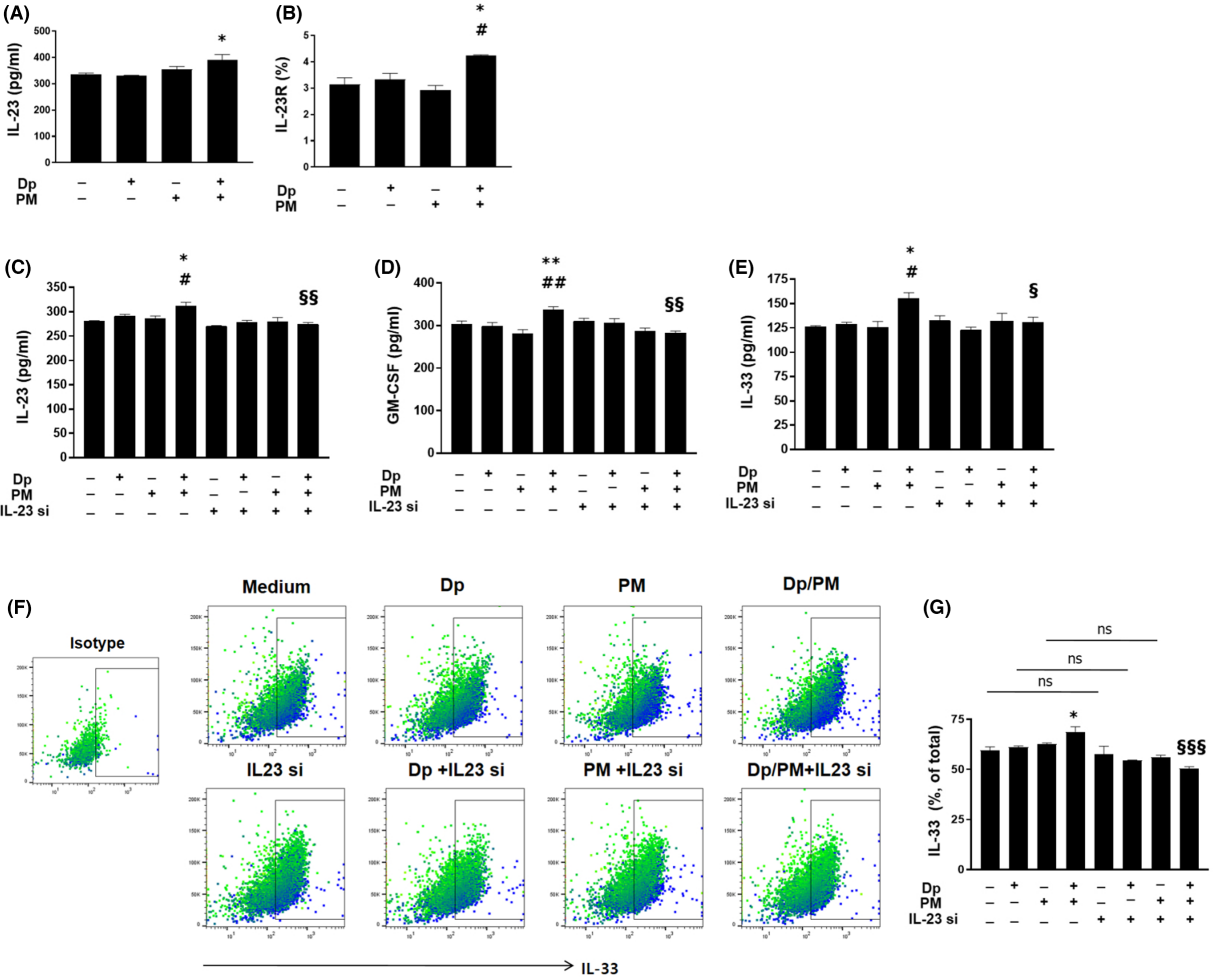
Expression of IL‐23, IL‐23R and the effect of IL‐23 inhibition in PM‐ and low‐dose Dp‐stimulated MLE‐12 cells. IL‐23 production from cytosol and IL‐23R expression in PM or low dose of Dp‐treated MLE12 cells (A). Expression of IL‐23R was analysed using flow cytometry (B). IL‐23, GM‐CSF in cytosol and IL‐33 production in nucleus (C‐E), the gating plot including isotype (647‐conjugated anti‐goat IgG) and frequency of intracellular IL‐33 using flow cytometry (F‐G) in PM or low dose of Dp‐stimulated MLE12 cells with or without IL‐23p19 siRNA or NC (Normal Control) siRNA. Data were analysed using flow cytometry. PM: Particulate matter. IL‐23R: IL‐23 Receptor, IL‐23 si: IL‐23p19 siRNA. Statistically significant (*P* < 0.05, *P* < 0.01, *P* < 0.001) differences from Dp group are represented by *, differences from PM group are represented by #, differences from Dp/PM group are represented by §. Statistical analysis followed by one‐way anova with Bonferroni's multiple comparisons test

First of all, we defined the stimulation of low concentrations of Dp in the experiments using airway epithelial cells of previously published studies.^15^, ^21^ We then noted that the cytosolic production of IL‐23 and surface expression of IL‐23R were significantly increased after stimulating MLE‐12 cells with PM and low‐dose Dp compared with low‐dose Dp stimulation only (Figure 5A,B). To further evaluate the role of IL‐23, we treated IL‐23 siRNA or NC siRNA in PM‐ and low‐dose Dp‐stimulated MLE‐12 cell. It was confirmed that IL‐23 expression was reduced by IL‐23 siRNA treatment (Figure 5C), and the protein levels of GM‐CSF and IL‐33 were significantly reduced by IL‐23 siRNA treatment compared to stimulation of PM and low‐dose Dp (Figure 5D,E). Additionally, the intracellular expression of IL‐33 was significantly reduced by IL‐23 siRNA treatment compared to stimulation of PM and low‐dose Dp (Figure 5F,G).

### 3.5 Blockade of IL‐23 in PM‐ and low‐dose Dp‐stimulated MLE‐12 cells suppressed the coexpression of IL‐13 and IL‐5 in ILC2s and the activation of BMDCs

**FIGURE 6:**
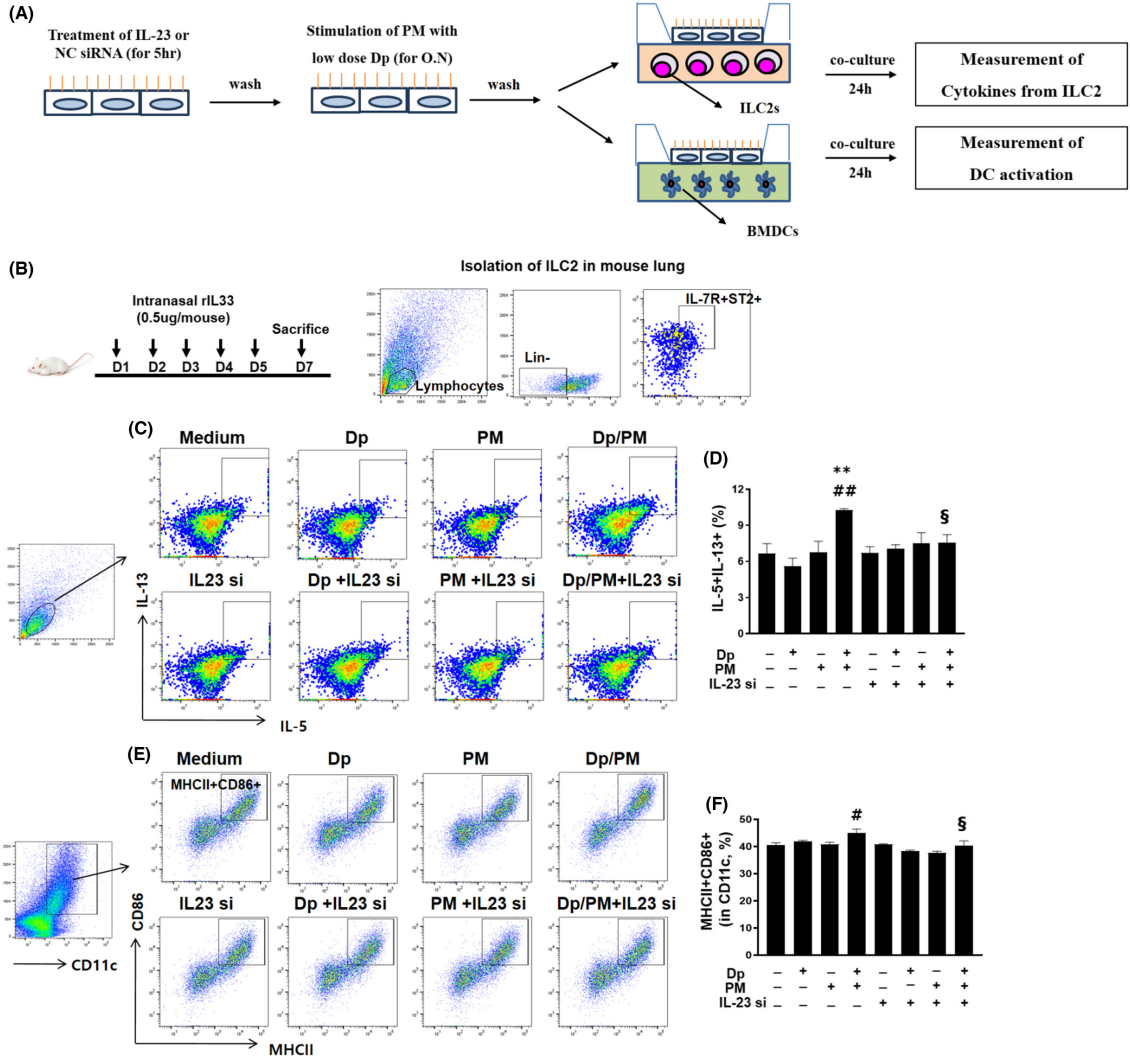
Coculture of IL‐23 siRNA pre‐treated and PM/low‐dose Dp‐stimulated MLE12 cells with ILC2 or BMDCs. Experimental procedure (A). The protocol for sorting ILC2 from IL‐33 administered mice (*n* = 8). For isolation of ILC2 from pooled mice, the population of ST2 + IL‐7R+ in lineage cells was sorted (B). For co‐cultivation, MLE‐12 cells were treated IL‐23p19 siRNA, and these MLE‐12 cells were stimulated by PM (0.1 μg/ml) or low dose of Dp (10 μg/ml) for overnight. After washing, cells were cocultured with ILC2. The gating plot and frequency of intracellular IL‐13 + IL‐5+ producing cells from isolated and expanded cells (C, D). For co‐cultivation with BMDCs, after treatment of IL‐23p19 siRNA in MLE‐12, PM or low dose of Dp was treated and after wash, then cocultured with BMDCs for 24 h. The gating plot and frequency of MHCII+CD86+ in CD11c+cells from BMDCs (E, F). PM: Particulate matter, IL‐23 si: IL‐23p19 siRNA. Statistically significant (*P* < 0.05, *P* < 0.01) differences from Dp group are represented by *, differences from PM group are represented by #, and differences from Dp/PM group are represented by §. Statistical analysis followed by one‐way anova with Bonferroni's multiple comparisons test

Finally, we determined whether the inhibition of IL‐23 in PM/low‐dose Dp‐stimulated MLE‐12 cells affected the expression of cytokines in ILC2s and the activation of BMDCs (Figure 6A). To sort ILC2s, we used several recombinant IL‐33‐administered mice. Pooled lung cells from mice were stained using the lineage cocktail, ST2 and IL‐7R antibodies (Figure 6B). We accordingly found that after coculture of IL‐23 siRNA‐pre‐treated and PM/low‐dose Dp‐stimulated MLE12 cells with ILC2s, the intracellular expression of IL‐13 and IL‐5 in ILC2s was significantly reduced compared with that in the PM/low‐dose Dp‐stimulated MLE12 cells (Figure 6C,D). Similarly, after coculture of IL‐23 siRNA pre‐treated and PM/low‐dose Dp‐stimulated MLE12 cells with BMDCs, the expression of MHCII and CD86 in CD11c from BMDCs was found to be significantly decreased compared with that in the PM/low‐dose Dp‐stimulated MLE12 cells (Figure 6E,F).

## 4 DISCUSSION

It is well known that PM can lead to various diseases.^22^ It has also been reported to be one of the most harmful factors involved in the occurrence and exacerbation of respiratory diseases.^23^ Epidemiological studies and animal experiments have also confirmed that elevated levels of PM can aggravate asthma.^24^ However, the relatively few mechanistic studies using animal models have been insufficient in indicating a causal relationship. On the one hand, exposure to PM, at high concentrations or prolonged time, can increase airway remodeling^25^ and proliferation of CD4 T cells by increasing GM‐CSF.^26^ Therefore, in this study, we determined the concentration of PM which did not cause an increase in Dp‐specific IgG1 and Th2 cytokines or induce hyper neutrophilia in the PM‐treated group. We also monitored changes in the asthma phenotype and immunologic response by administering low‐dose Dp alone; no significant changes were observed. In this study, we demonstrated that exposure to PM and low‐dose Dp leads to an increase in AHR, allergic responses, populations of Th2, Th17 and ILC2 cells.

Similarly, in recent animal studies on PM and asthma, simultaneous exposure to DEP and HDM,^27^ as well as exposure of OVA‐administered mice to PM,^28^ have been shown to increase the population of Th2 and Th17 cells, which are adaptive immune responses, as a major response mechanism. In the present study, we also confirmed that the numbers of Th2 and Th17 cells were significantly increased following exposure to PM and low‐dose Dp compared with those in the PM and Dp alone groups.

The innate immune response also plays a significant role in the induction and aggravation of asthma.^29^ It has been demonstrated that in DEP and HDM co‐exposed mice, IL‐33 levels are also increased leading to amplified ILC2 population; the results of experiments using ST2‐null mice suggested that ILC2 played a partial role in promoting airway hypersensitivity.^7^ Consistent with this finding, we confirmed that ILC2 was increased after administration of PM and low‐dose Dp. However, we did not observe a significant increase in ILC3, which suggests that ILC3 population did not increase even in the recently reported DEP‐ and HDM‐exposed mice.^8^ Therefore, we considered that ILC2 plays a critical role in the mechanism of enhancement of PM‐mediated allergic asthma.

IL‐23 is the principal cytokine secreted by antigen‐presenting cells known to induce acquired immune responses in asthma; however, it has also been reported to play a role in innate immune responses as well as adaptive immune responses.^21^ IL‐23 is also expressed in airway epithelial cells and has been suggested that it acts in an autocrine manner.^14^, ^21^ Therefore, We hypothesise that IL‐23 contributes to an increased allergic response upon exposure to PM and low‐dose Dp. We confirmed the increase in the expression of IL‐23 in epithelial cells (Epcam+CD45−) and CD11c+ cells when exposed to PM and low‐dose Dp; the expression of IL‐23R was also increased in epithelial cells (Epcam+CD45−). In the next experiment, we observed that intranasal administration of anti‐IL‐23 Ab decreased allergic reactions and adaptive immune responses following exposure to PM and low‐dose Dp, and also led to a significant decrease in ILC2 populations. Innate cytokines secreted from epithelial cells can aggravate allergic responses, among which IL‐33 and GM‐CSF play a major role in ILC2 and DC activation.^30^, ^31^

In the result of our experiment, a decrease in IL‐33, GM‐CSF and IL‐1α expression was confirmed in lung tissue on inhibition of IL‐23. Based on the confirmation of increased expression of IL‐23 and IL‐23R in airway epithelial cells in this mouse study, we attempted to prove the hypothesis through an airway epithelial cell line experiment. It was found that IL‐23, secreted from airway epithelial cells during PM with low‐dose Dp stimulation, enhanced the expression changes of IL‐33 and GM‐CSF, and contributed to ILC2 and BMDC activation through in vitro coculture experiment. Therefore, as the key mechanism for increased allergic response, it is considered that induced changes of the expression of IL‐33 and GM‐CSF by IL‐23 can be important.

In recent study presented by this research group,^21^ we suggested that the expression of IL‐33, GM‐CSF and IL‐1α was significantly decreased when IL‐23 or IL‐23R was inhibited in an antigen‐stimulated airway epithelial cell line. It was also confirmed that AHR, eosinophils, Th2 and ILC2 cells occurred owing to the inhibition of IL‐1α. Other studies have also reported that IL‐1α, secreted by airway epithelial cells, controlled allergic sensitization via the epithelial release of GM‐CSF and IL‐33 on the inhalation of HDMs.^32^

In present study, we also confirmed the reduction of IL‐1α levels in lung tissues of anti‐IL‐23 Ab‐treated mice, when exposed to PM and low‐dose Dp. Therefore, these cytokines are considered crucial for increased innate and adaptive immune responses in Dp‐induced allergic asthma. The pathway associated with induction of IL‐33, GM‐CSF and IL‐1α may get influenced by PM and low‐dose Dp, regardless of the changes in IL‐23 levels. However, in this study, we sought to identify upstream targets that could regulate the expression of all the above‐mentioned cytokines (IL‐33, GM‐CSF and IL‐1α). The present results confirmed that IL‐33, GM‐CSF and IL‐1α were all elevated when PM and low‐dose Dp were administered to the mice, but decreased when IL‐23 was inhibited. Therefore, IL‐23 can be an effective therapeutic target for inhibiting allergic reactions in airway epithelial cells in asthma. Although the mechanism through which IL‐23 regulates IL‐33, GM‐CSF and IL‐1α is unclear, it can be elucidated in future experiments. It is also unclear whether IL‐23, secreted by bronchial epithelial cells, directly or upstream regulates IL‐33, GM‐CSF and IL‐1α; this can also be confirmed by future experiments.

Meanwhile, the barrier dysfunction of airway epithelial cells also plays a role in increasing their susceptibility to antigens by enhancing the penetration of inhaled harmful substances.^33^ Although not measured in this experiment, direct DC activation by Dp can occur through physical changes in epithelial cells following PM and Dp administration.

CD4+ T cells play an important role in the pathophysiology of asthma.^34^ It is also known that an increase in DC activation can induce activation and differentiation of CD4+ T cells.^35^, ^36^

The secretion of IL‐23 by DC increases on aggravated DC activation.^37^ Moreover, IL‐23 secreted from DCs has an autocrine action and contributes to further DC activation.^13^ However, there was no change in IL‐23R expression in DC was observed in the PM‐ and low‐dose Dp‐administered mice in the present study. Various studies have stated that IL‐23 secreted by DC plays a significant role, not only in increasing Th17 cells but also Th2 cells,^38^ Therefore, DC and CD4 T‐cell interactions may have played a role in the mechanism of the present study. Our study attempted to focus on the role of PM‐ and low‐dose Dp‐induced increase in IL‐23 levels in airway epithelial cells in association with innate immunity, which is still unknown, and to prove whether it affects ILC2 as well as DC. Therefore, to confirm the role of CD4 T cells, future studies on DC and CD4 T cell coculture experiments could be beneficial.

IL‐23 is also known to increase ILC3^29^; however, no significant increase in ILC3 was observed in the PM with low‐dose Dp in this study.

IL‐13 and IL‐17A promote increased α‐SMA expression.^39^, ^40^ The present result also shows change in α‐SMA expression suggesting it as one of the mechanisms influenced by AHR. Recently, an increase in dual‐positive Th2/Th17 cells, including IL‐13+ IL‐17A+ double‐producing T cells, was suggested as a cause of increased severity in patients with severe asthma^19^ with synergestic exposure of DEP with HDM.^8^, ^27^ In the present study, the population of IL‐13+ in IL‐17+ CD4 cells from lung tissues was increased in PM with low‐dose Dp‐administered mice. The population of IL‐5+ in IL‐17+ CD4 cells was small, but also increased in mice administered PM with low‐dose Dp. However, these cell populations were significantly reduced by IL‐23 inhibition. Although the role of each IL‐13+ or IL‐5+ population in IL‐17+ CD4 cells was observed in the study, the mechanism of changes in dual‐positive Th2/Th17 populations through IL‐23 inhibition should be studied in the future; based on our knowledge, this is the first report of changes in these populations caused by IL‐23 inhibition. The increase in dual‐positive Th2/Th17 population due to IL‐23 suggests an important role in the mechanism of severe asthma.

**FIGURE 7:**
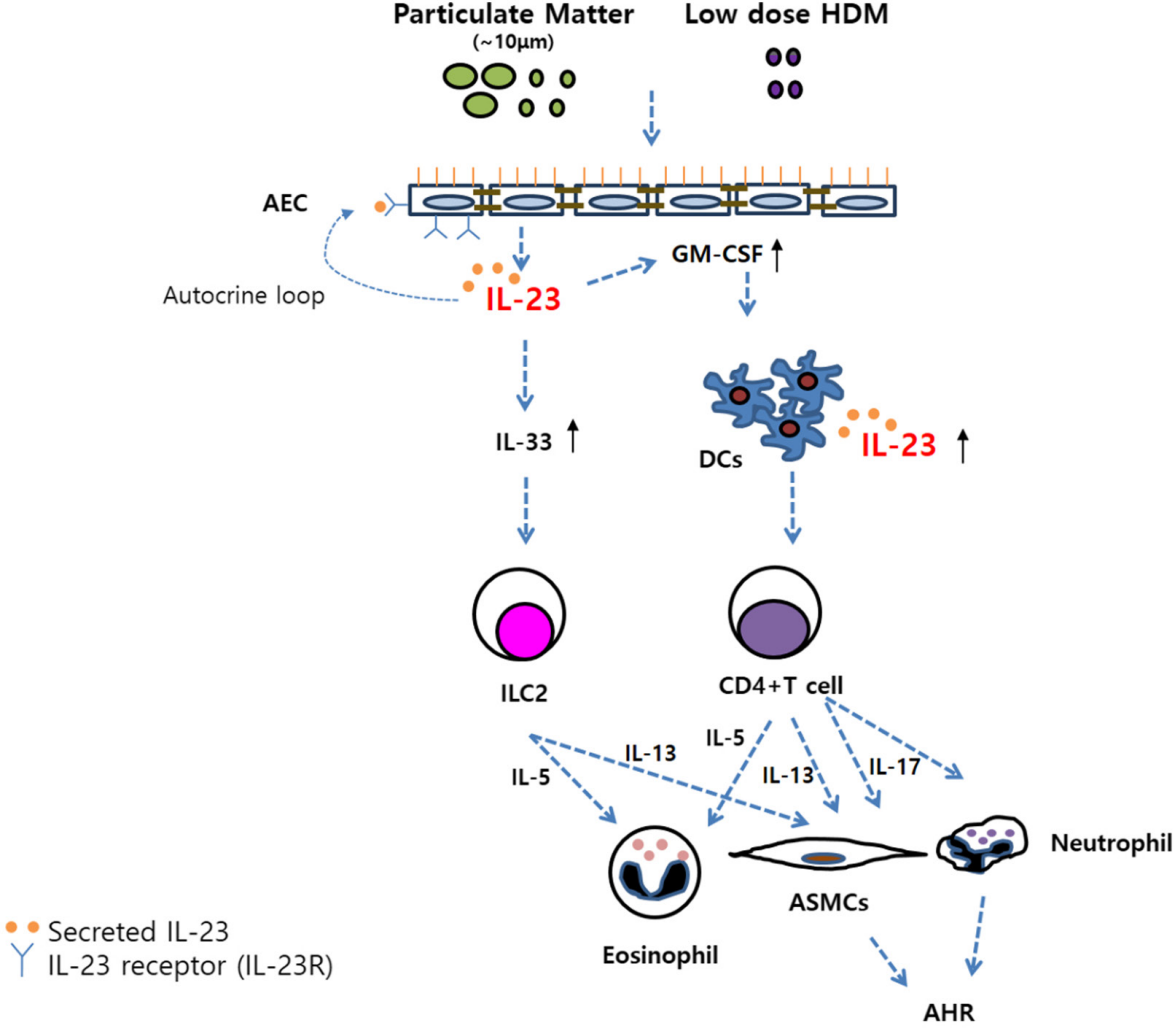
Possible role of IL‐23 in particulate matter‐mediated allergic asthma. Exposure of particulate matter (PM) and low dose of HDM induces IL‐23 secretion, IL‐23R expression and IL‐33 production in airway epithelial cells. Increased IL‐23 also binds to IL‐23R in airway epithelial cells. These can also enhance IL‐33 production and activate ILC2s. Activated ILC2s causes eosinophilic airway inflammation and AHR. In addition, increased IL‐23 from airway epithelial cells increase the production of GM‐CSF. The increment of GM‐CSF leads to DCs activation, and exposure of particulate matter (PM) and low dose of HDM can also increase the expression of IL‐23 from DCs. Increased IL‐23 from activated DCs induce activation of Th2 cytokines and IL‐17 in CD4 T cells. Activated Th2 cytokines and IL‐17 in CD4 T cells cause eosinophilic airway inflammation and AHR, due to increased α‐smooth muscle actin (α‐SMA) and neutrophils. AEC, airway epithelial cell; AHR, airway hyperresponsiveness; ASMC, airway smooth muscle cell; HDM, house dust mite

In this study, we postulated that PM augments the phenotypes of allergic asthma upon exposure to low‐dose Dp, and immune responses such as ILC2, Th2 cells, Th17 cells, IL13+ or IL‐5+ population from IL17+ CD4+ cells were all increased; however, inhibition of IL‐23 can reduce these phenotypes and immunologic changes. IL‐23 secreted by airway epithelial cells contributes to the augmentation of immunologic changes by co‐exposure of PM and low‐dose Dp (Figure 7).

## AUTHOR CONTRIBUTIONS

**Hyun Seung Lee:** Conceptualization (lead); data curation (lead); formal analysis (lead); funding acquisition (lead); investigation (equal); methodology (equal); project administration (lead); supervision (equal); validation (equal); writing – original draft (lead); writing – review and editing (equal). **Heung‐Woo Park:** Conceptualization (equal); data curation (equal); formal analysis (equal); investigation (equal); methodology (equal); validation (equal); writing – original draft (equal); writing – review and editing (equal).

## CONFLICT OF INTEREST

The authors have no competing interests to declare.

## Supporting information


Appendix S1

## Data Availability

All data have been reported.
