# Supplementary material for: IL‐23 plays a significant role in the augmentation of particulate matter‐mediated allergic airway inflammation
Source: J Cell Mol Med. 2022 Jul 8;26(16):4506–19. doi: 10.1111/jcmm.17475 (PMC9357615; doi:10.1111/jcmm.17475)
Supplement: Supplementary file 1 — Appendix S1 [file JCMM-26-4506-s001.doc]

**IL-23 plays a significant role in the augmentation of particulate matter-mediated allergic airway inflammation**

Hyun Seung Lee^1^, Heung-Woo Park^2,3^

^1^Biomedical Research Institute, Seoul National University Hospital, Seoul, Republic of Korea

^2^Department of Internal Medicine, Seoul National University Hospital, Seoul, Republic of Korea

^3^Department of Internal Medicine, Seoul National University College of Medicine, Seoul, Republic of Korea

**Corresponding author:**

Hyun Seung Lee, PhD

Biomedical Research Institute, Seoul National University Hospital, 101 Daehak-ro, Jongno-gu, Seoul, 110-744, Korea

Fax: +82-2-742-3291, Tel: +82-2-2072-4409, E-mail: [gentlehood79@ hanmail.net](mailto:gentlehood79@hanmail.net)

**Supplementary Figure**

**
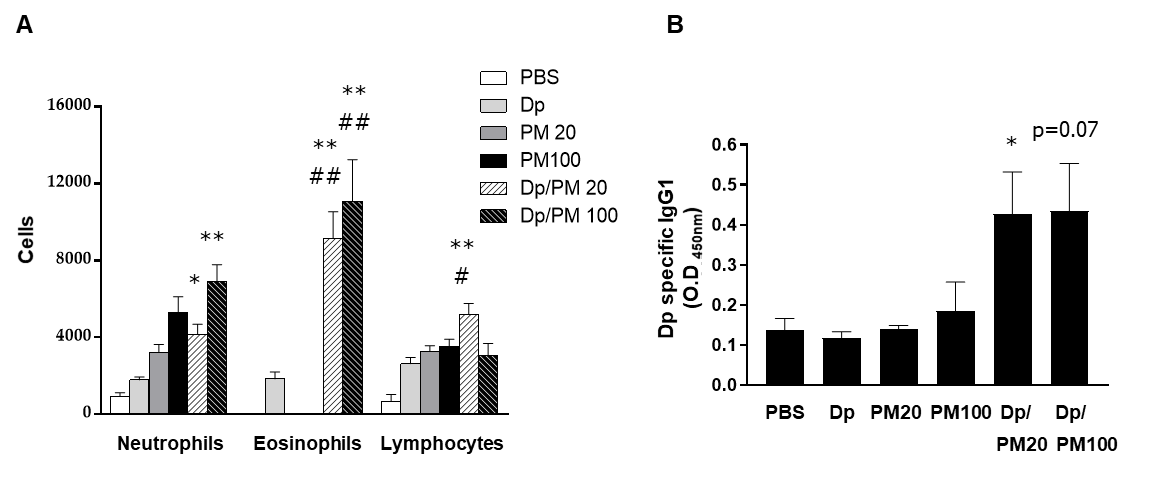
**


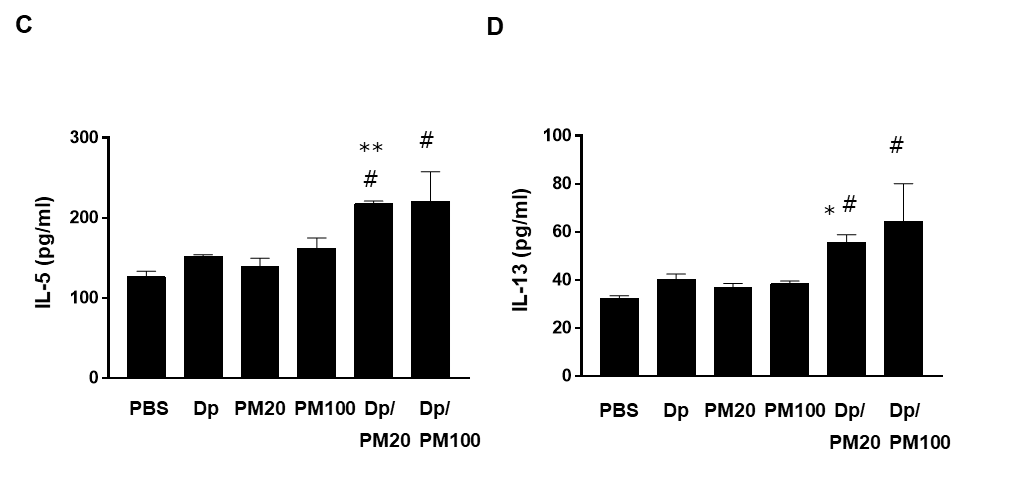


**Fig. S1. The evaluation of changes in PM and low dose Dp exposed mice**

PM 20 or 100 μg with low dose of Dp (10 μg) were instillated in mice on Day 1,3,5,8,10,12 and 15 (4-5 mice in each group). 24h after the last instillation, changes were evaluated. The numbers of inflammatory cells in BALF (A). Serum Dp-specific IgG1 (B). The protein level of IL-5 and IL-13 in BALF was detected using ELISA (C-D). PM; Particulate Matter. Statistically significant (P<0.05, P<0.01) differences from Dp group are represented by *, and differences from each dose of PM group are represented by #. Statistical analysis followed by one-way ANOVA with Bonferroni’s multiple comparisons test.
